# Supplementary material for: A cell atlas of the larval Aedes aegypti ventral nerve cord
Source: Neural Dev. 2024 Jan 31;19:2. doi: 10.1186/s13064-023-00178-8 (PMC10829479; doi:10.1186/s13064-023-00178-8)

## Figure S1

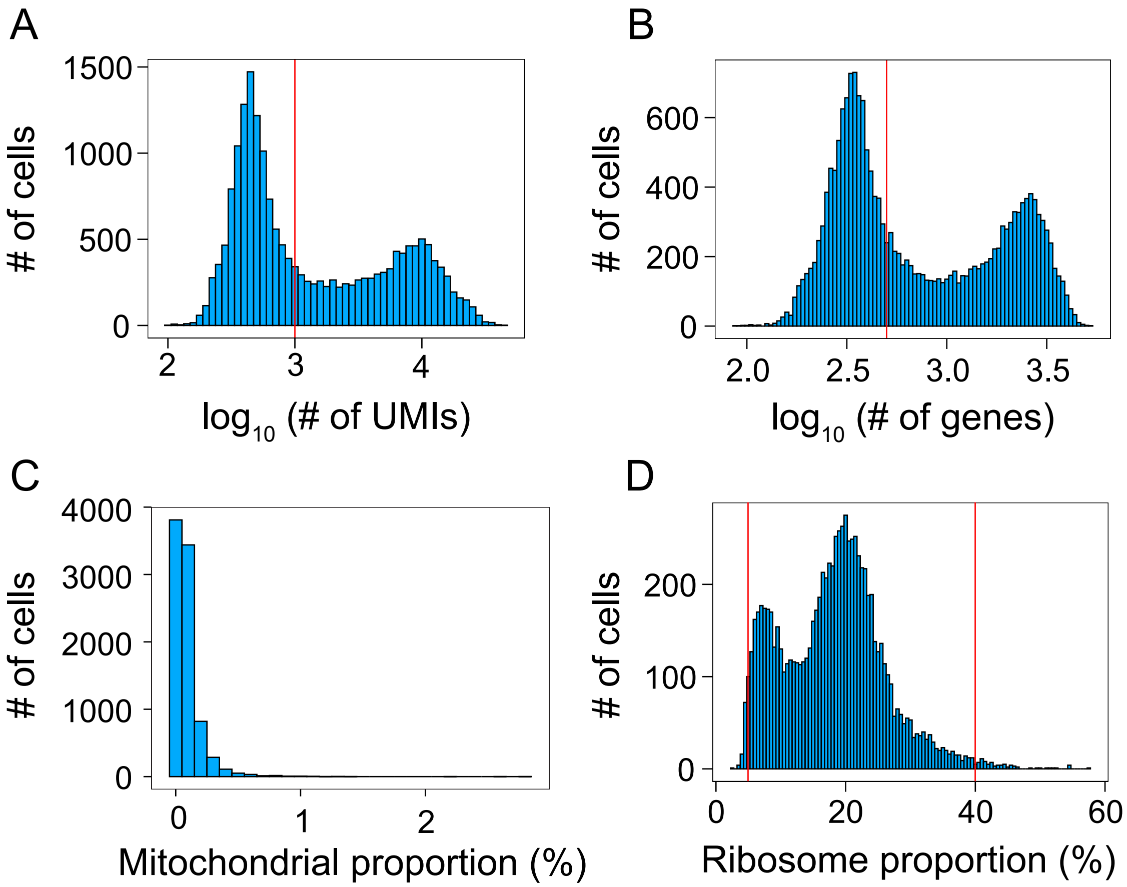

A
1500
1000
500
0
# of cells
2
3
4
log10 (# of UMIs)
B
600
400
200
0
# of cells
2.0
2.5
3.0
3.5
log10 (# of genes)
C
4000
3000
2000
1000
0
# of cells
0
1
2
Mitochondrial proportion (%)
D
200
100
0
# of cells
0
20
40
60
Ribosome proportion (%)

**Figure S2**

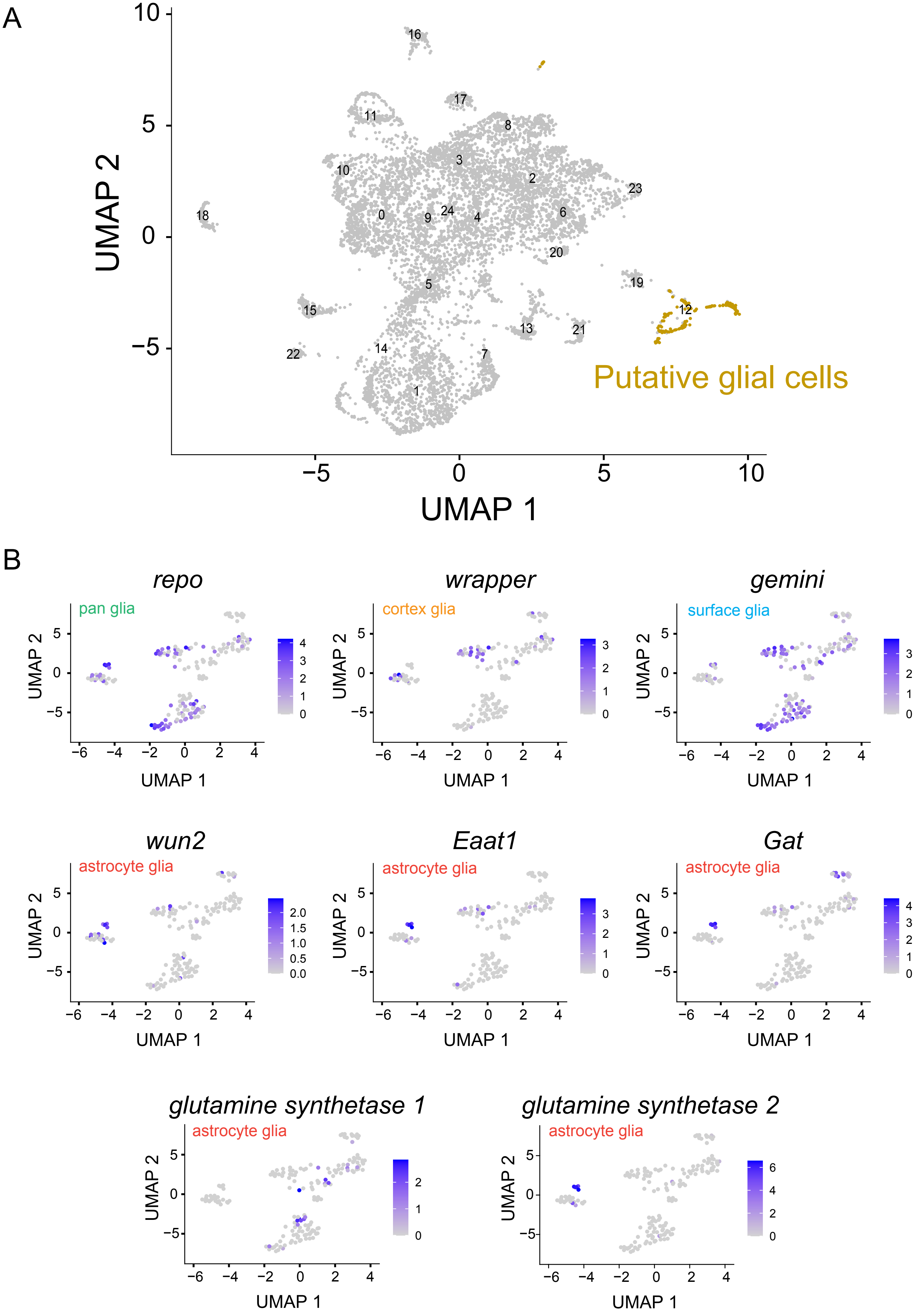

A
UMAP 2
UMAP 1
16
17
11
8
3
10
2
23
18
0
9
24
4
6
20
5
19
15
12
13
21
22
14
7
1
Putative glial cells
B
repo
pan glia
wrapper
cortex glia
gemini
surface glia
wun2
astrocyte glia
Eaat1
astrocyte glia
Gat
astrocyte glia
glutamine synthetase 1
astrocyte glia
glutamine synthetase 2
astrocyte glia

**Figure S3**

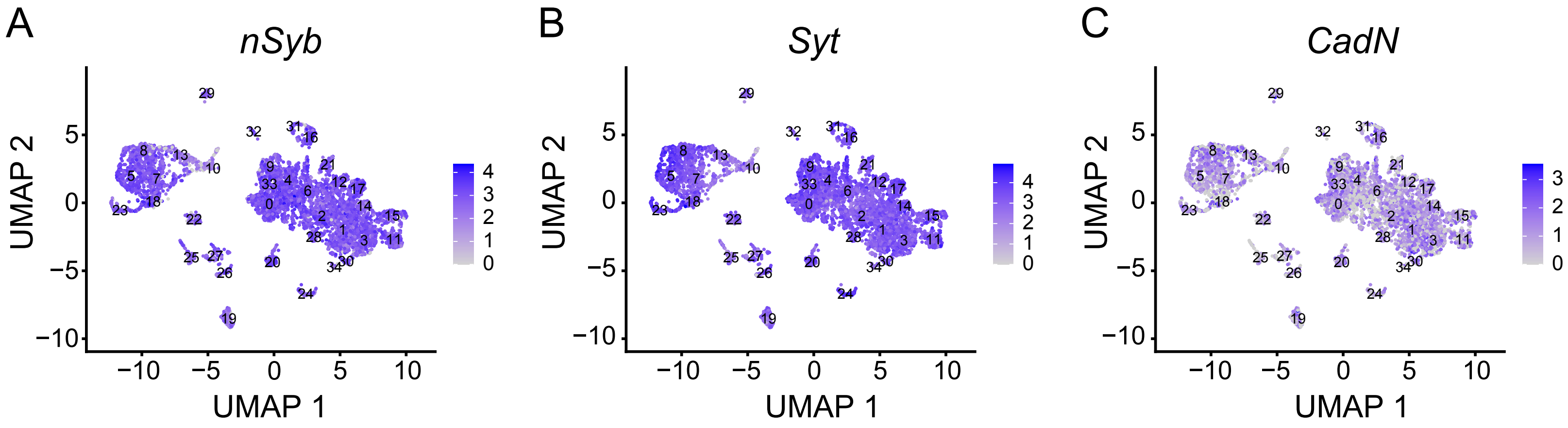

# Figure S4

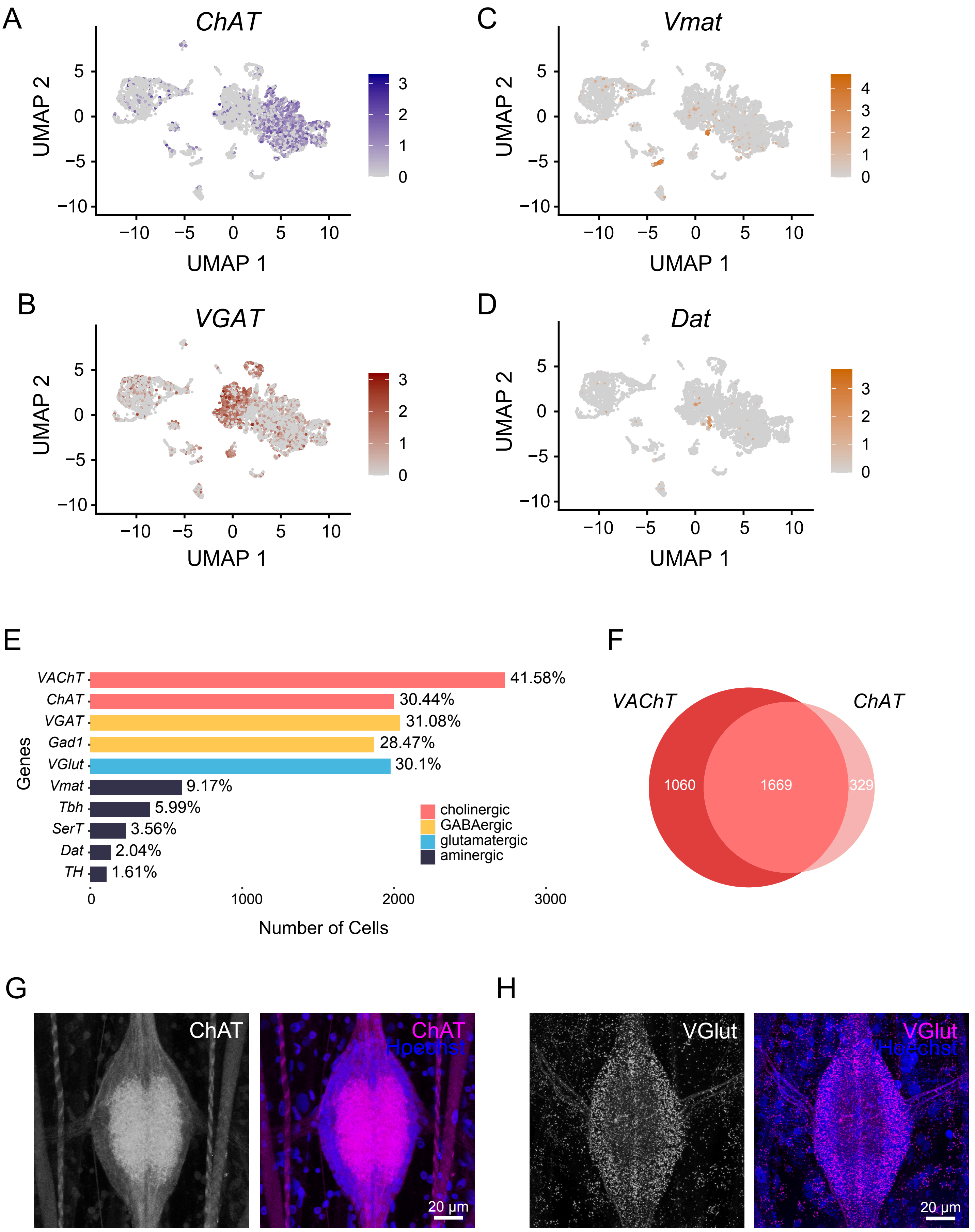
A
ChAT
UMAP 2
UMAP 1
B
VGAT
C
Vmat
D
Dat
E
VAChT
ChAT
VGAT
Gad1
VGlut
Vmat
Tbh
SerT
Dat
TH
41.58%
30.44%
31.08%
28.47%
30.1%
9.17%
5.99%
3.56%
2.04%
1.61%
Genes
0
1000
2000
3000
Number of Cells
cholinergic
GABAergic
glutamatergic
aminergic
F
VAChT
ChAT
1060
1669
329
G
ChAT
ChAT
Hoechst
20 µm
H
VGlut
VGlut
/Hoechst
20 µm

**Figure S5**

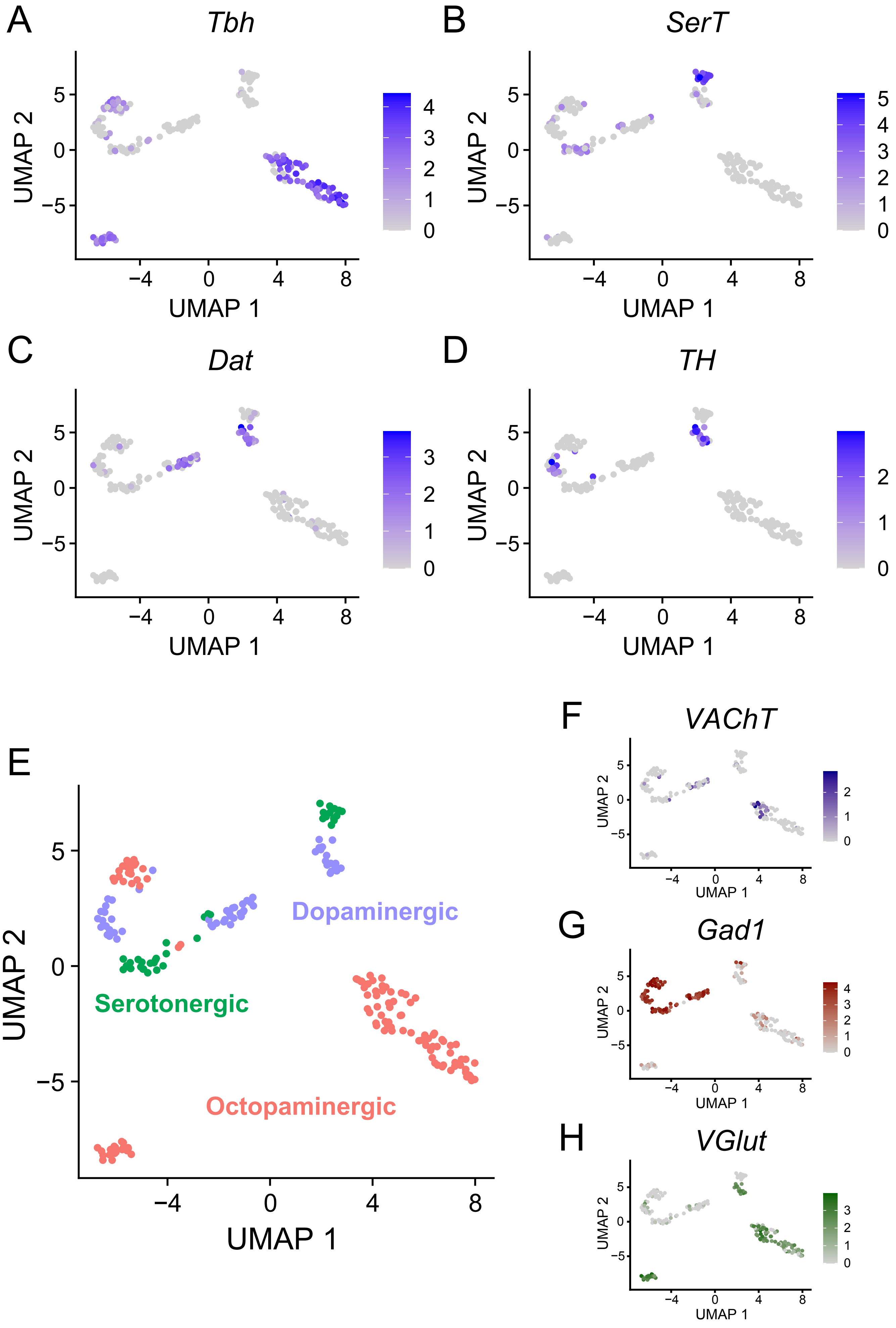
A
Tbh
B
SerT
C
Dat
D
TH
E
Dopaminergic
Serotonergic
Octopaminergic
F
VAChT
G
Gad1
H
VGlut
UMAP 1
UMAP 2

## Figure S6

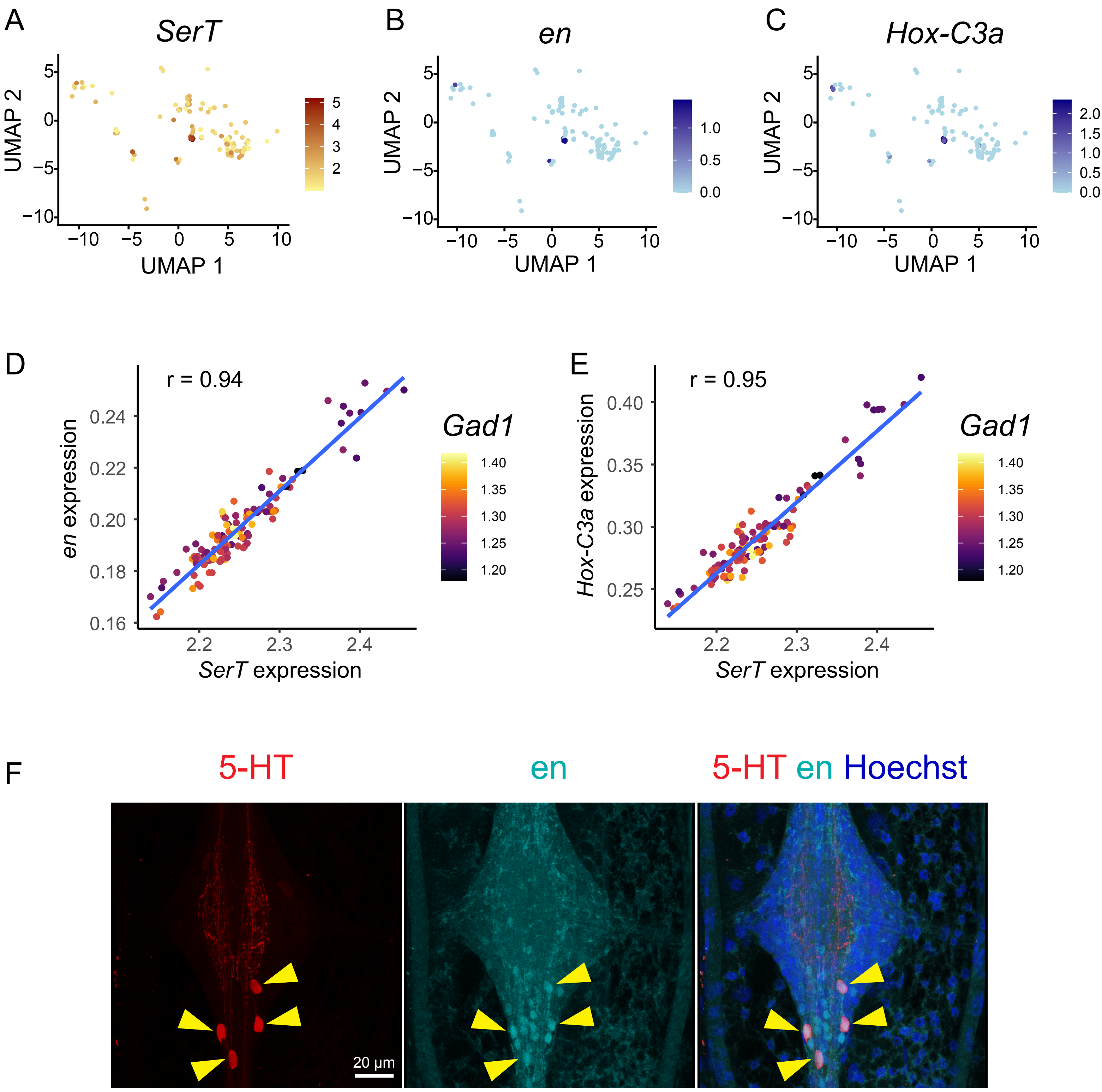
A
SerT
UMAP 2
UMAP 1
B
en
C
Hox-C3a
D
r = 0.94
en expression
SerT expression
Gad1
E
r = 0.95
Hox-C3a expression
F
5-HT
en
5-HT en Hoechst
20 µm

# Figure S7

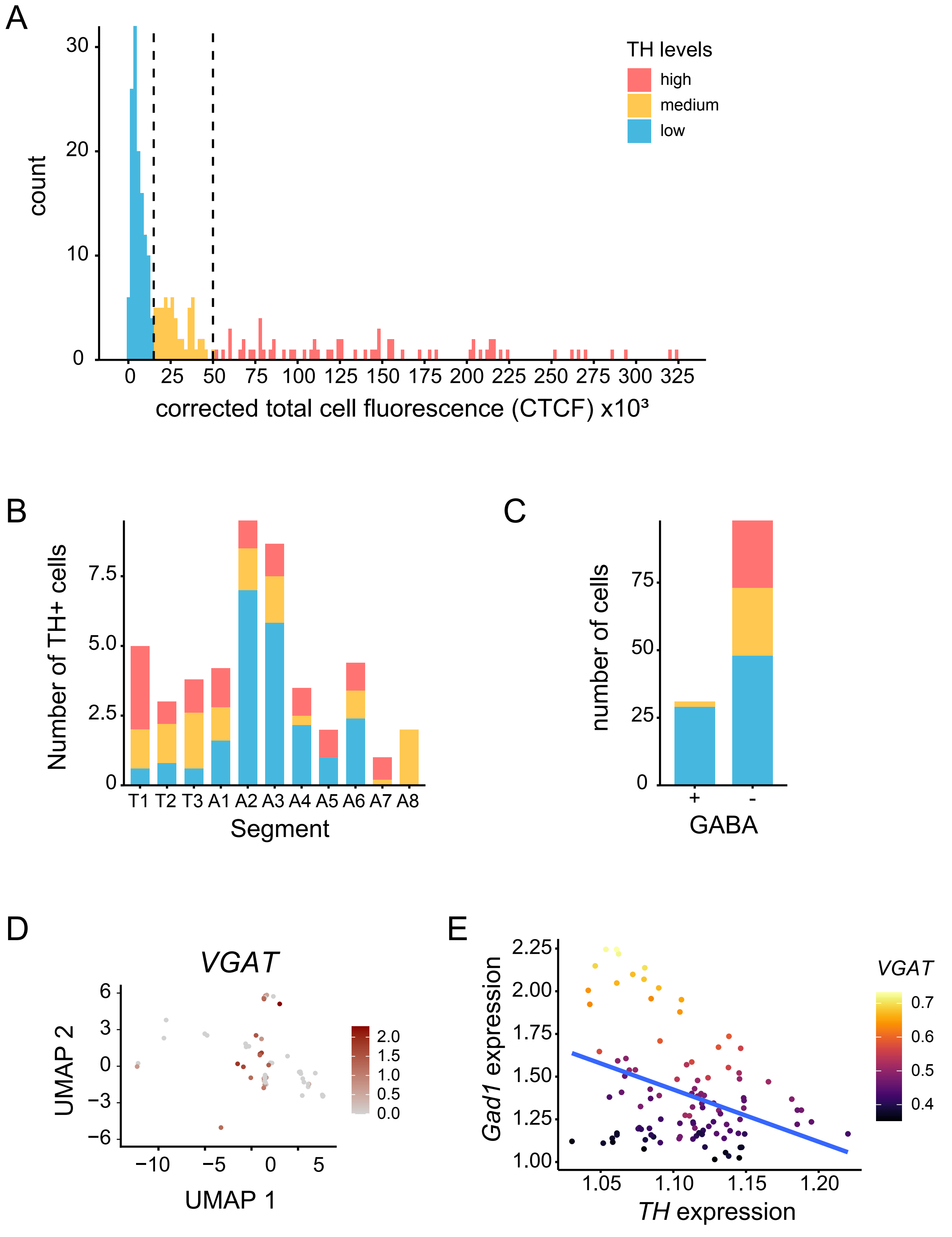

A
TH levels
high
medium
low
count
30
20
10
0
0 25 50 75 100 125 150 175 200 225 250 275 300 325
corrected total cell fluorescence (CTCF) x10³
B
Number of TH+ cells
7.5
5.0
2.5
0
T1 T2 T3 A1 A2 A3 A4 A5 A6 A7 A8
Segment
C
number of cells
75
50
25
0
+
-
GABA
D
VGAT
UMAP 2
6
3
0
−3
−6
−10 −5 0 5
UMAP 1
2.0
1.5
1.0
0.5
0.0
E
Gad1 expression
2.25
2.00
1.75
1.50
1.25
1.00
1.05 1.10 1.15 1.20
TH expression
VGAT
0.7
0.6
0.5
0.4

# Figure S8

A

## *Neuropeptides*

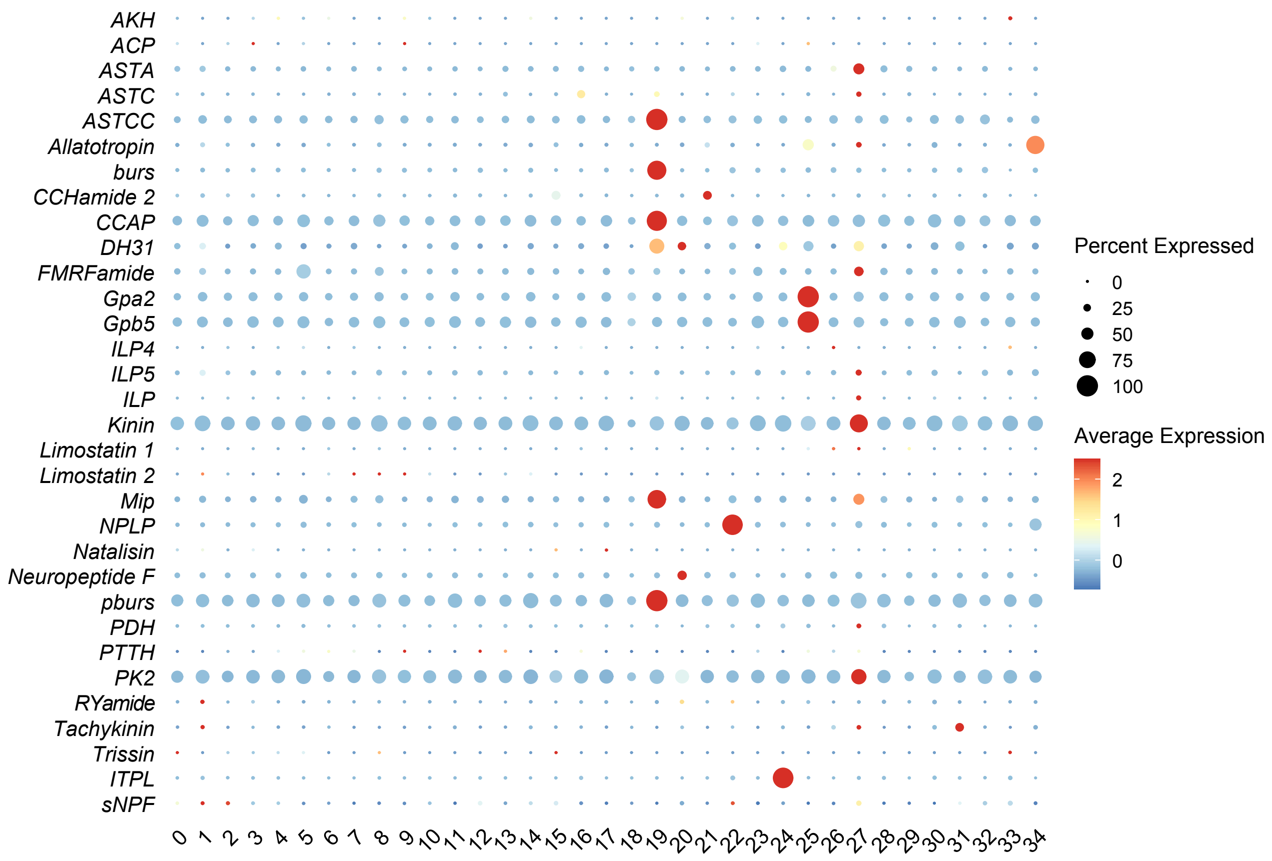


B

## *Peptide processing enzymes*

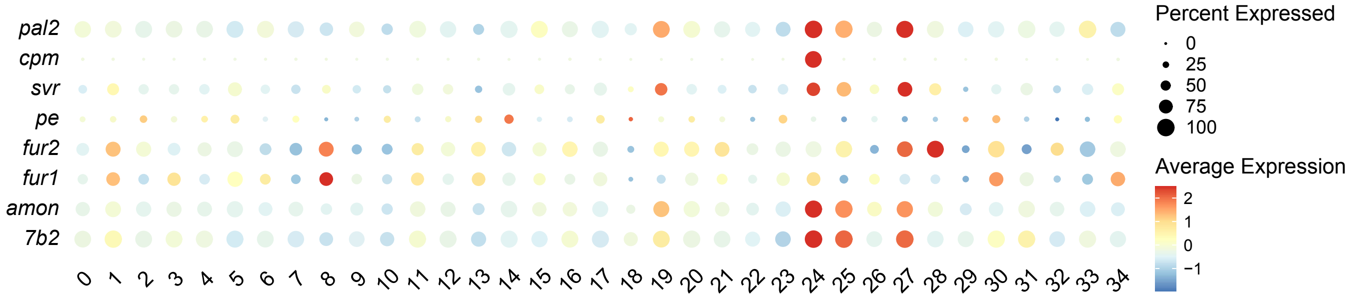

# Figure S9

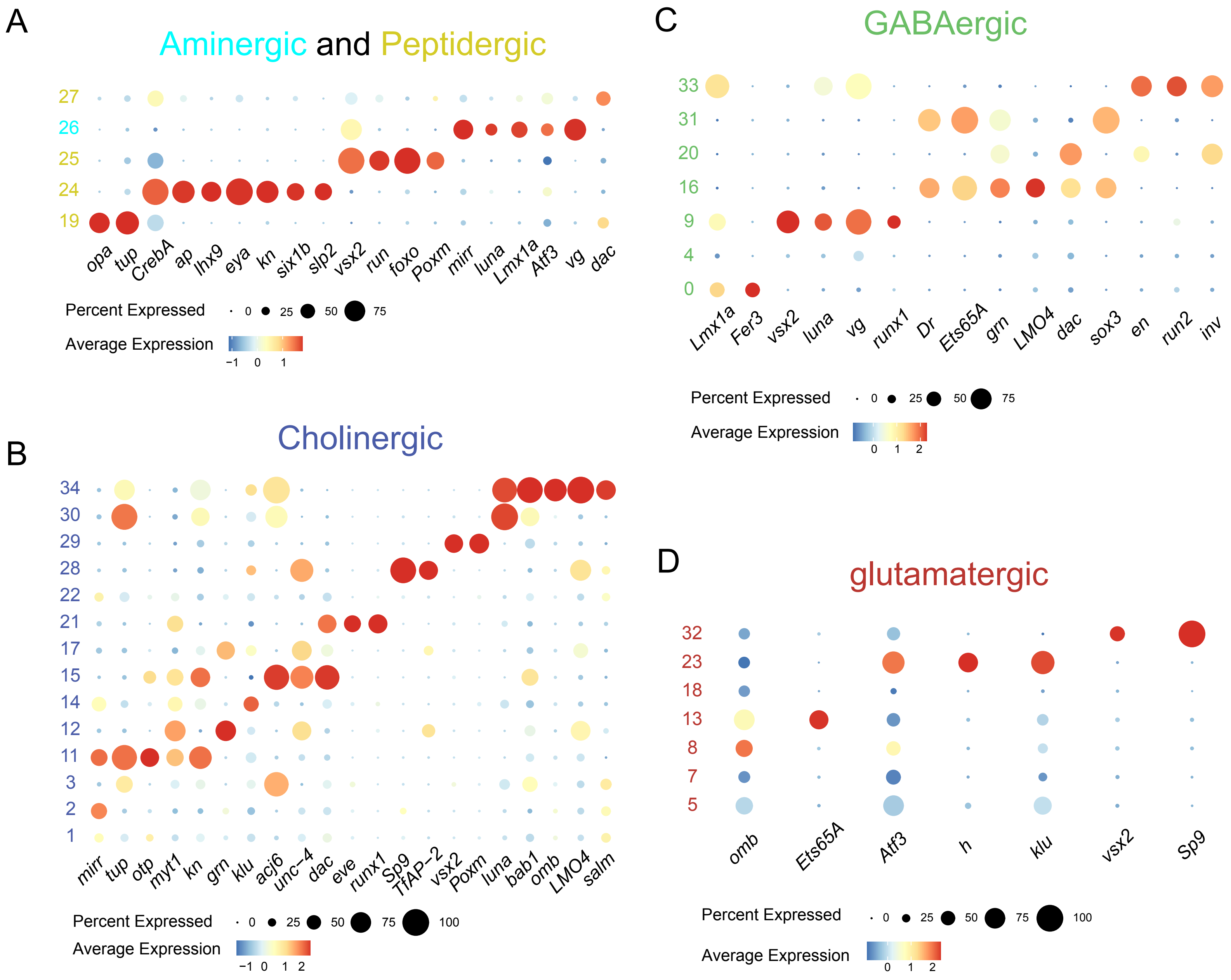
A
Aminergic and Peptidergic
27
26
25
24
19
opa tup CrebA ap lhx9 eya kn six1b slp2 vsx2 run foxo Poxm mirr luna Lmx1a Atf3 vg dac
Percent Expressed 0 25 50 75
Average Expression −1 0 1
C
GABAergic
33
31
20
16
9
4
0
Lmx1a Fer3 vsx2 luna vg runx1 Dr Ets65A grn LMO4 dac sox3 en run2 inv
Percent Expressed 0 25 50 75
Average Expression 0 1 2
B
Cholinergic
34
30
29
28
22
21
17
15
14
12
11
3
2
1
mirr tup otp myt1 kn grn klu acj6 unc-4 dac eve runx1 Sp9 TfAP-2 vsx2 Poxm luna bab1 omb LMO4 salm
Percent Expressed 0 25 50 75 100
Average Expression −1 0 1 2
D
glutamatergic
32
23
18
13
8
7
5
omb Ets65A Atf3 h klu vsx2 Sp9
Percent Expressed 0 25 50 75 100
Average Expression 0 1 2

# Figure S10

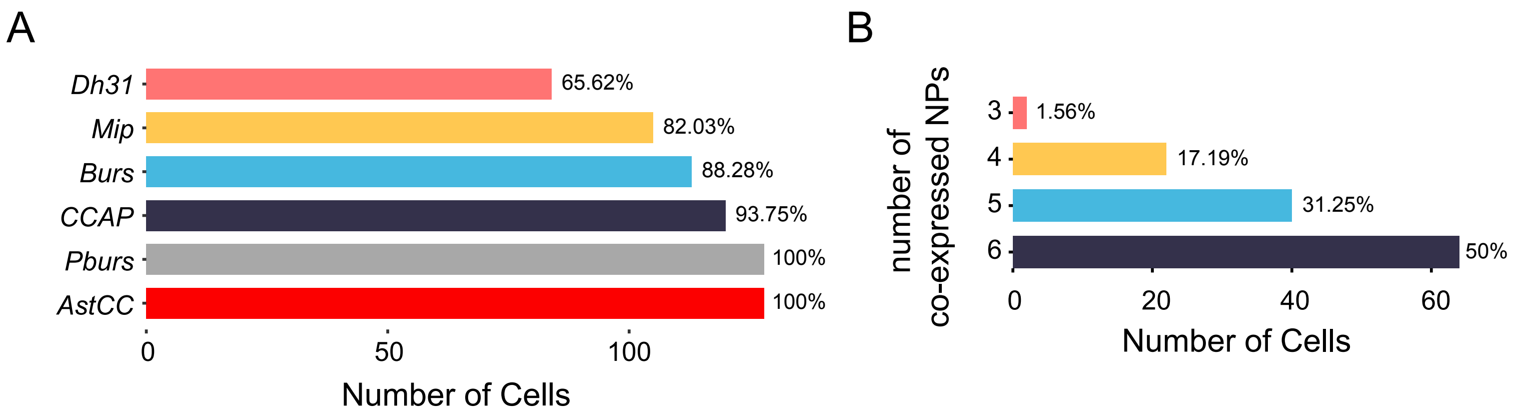
A
Dh31
Mip
Burs
CCAP
Pburs
AstCC
65.62%
82.03%
88.28%
93.75%
100%
100%
0
50
100
Number of Cells
B
number of
co-expressed NPs
3
4
5
6
1.56%
17.19%
31.25%
50%
0
20
40
60
Number of Cells

## Figure S11

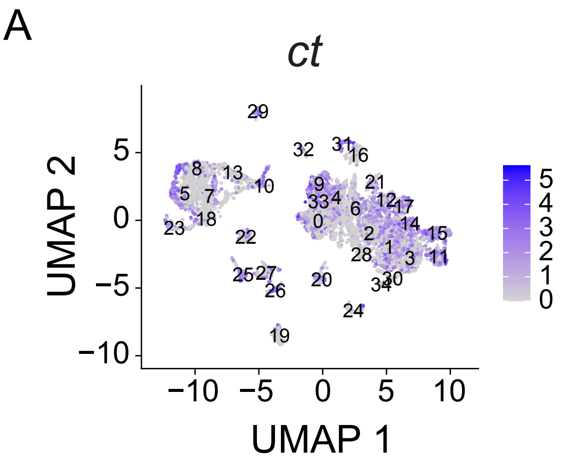
A
ct
UMAP 2
UMAP 1
−10
−5
0
5
10
29
32
31
16
8
13
10
5
7
23
18
22
25
27
26
19
20
24
9
33
4
0
6
21
12
17
14
15
2
1
28
3
11
34
30
5
4
3
2
1
0

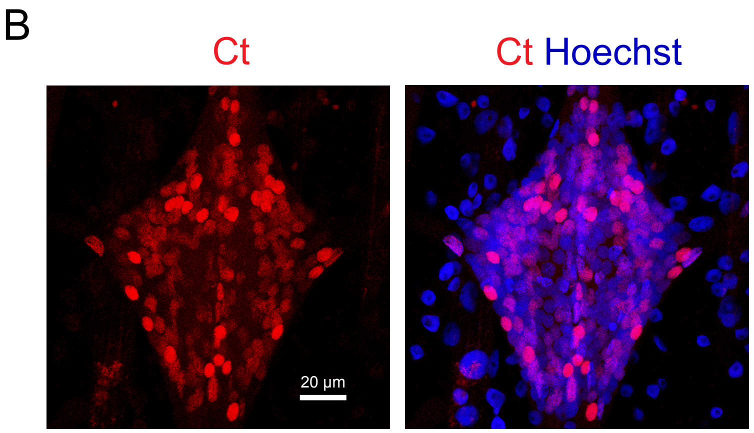
B
Ct
Ct Hoechst
20 µm

# Figure S12

A

## FAN receptors

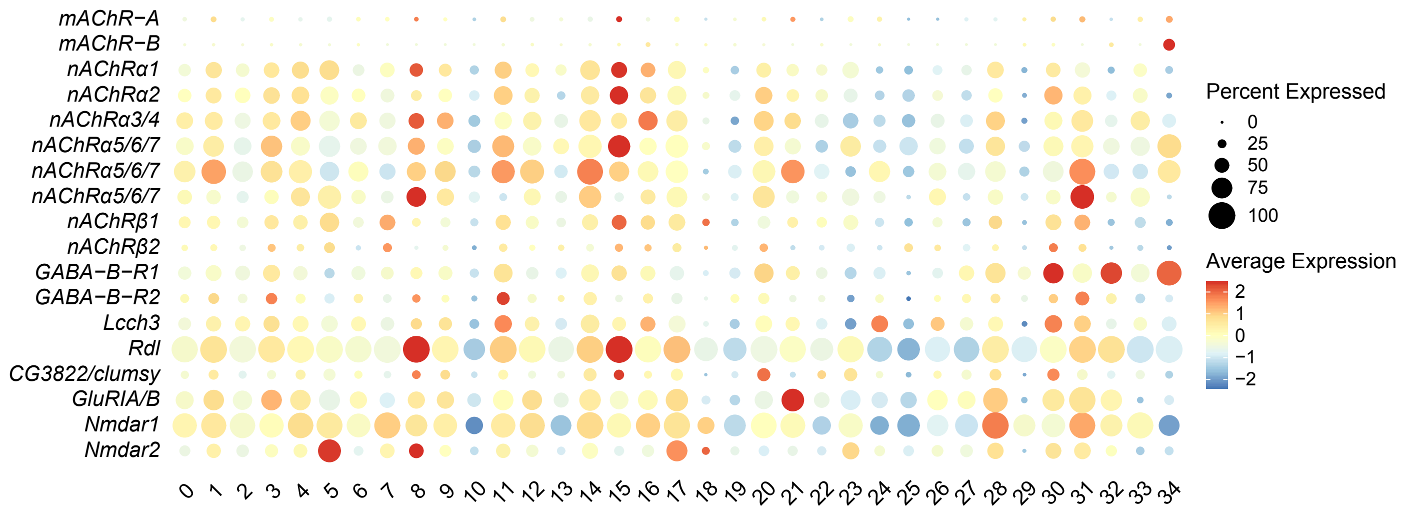


B

## monoamine receptors

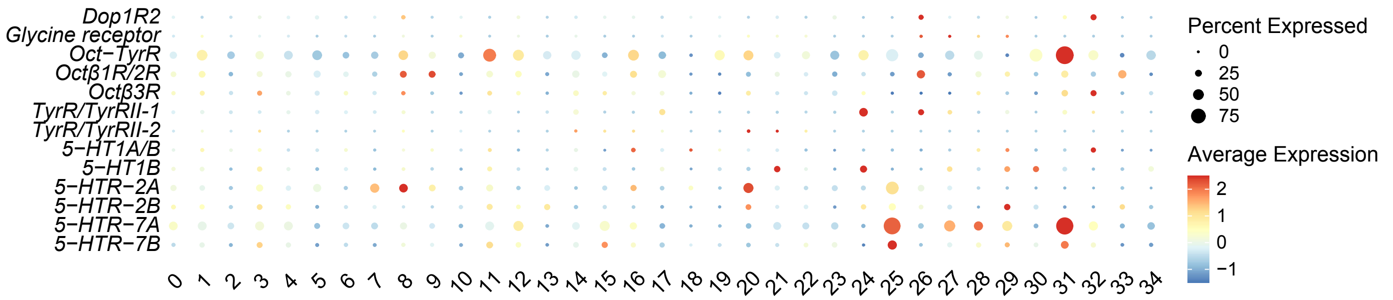


C

## neuropeptide receptors

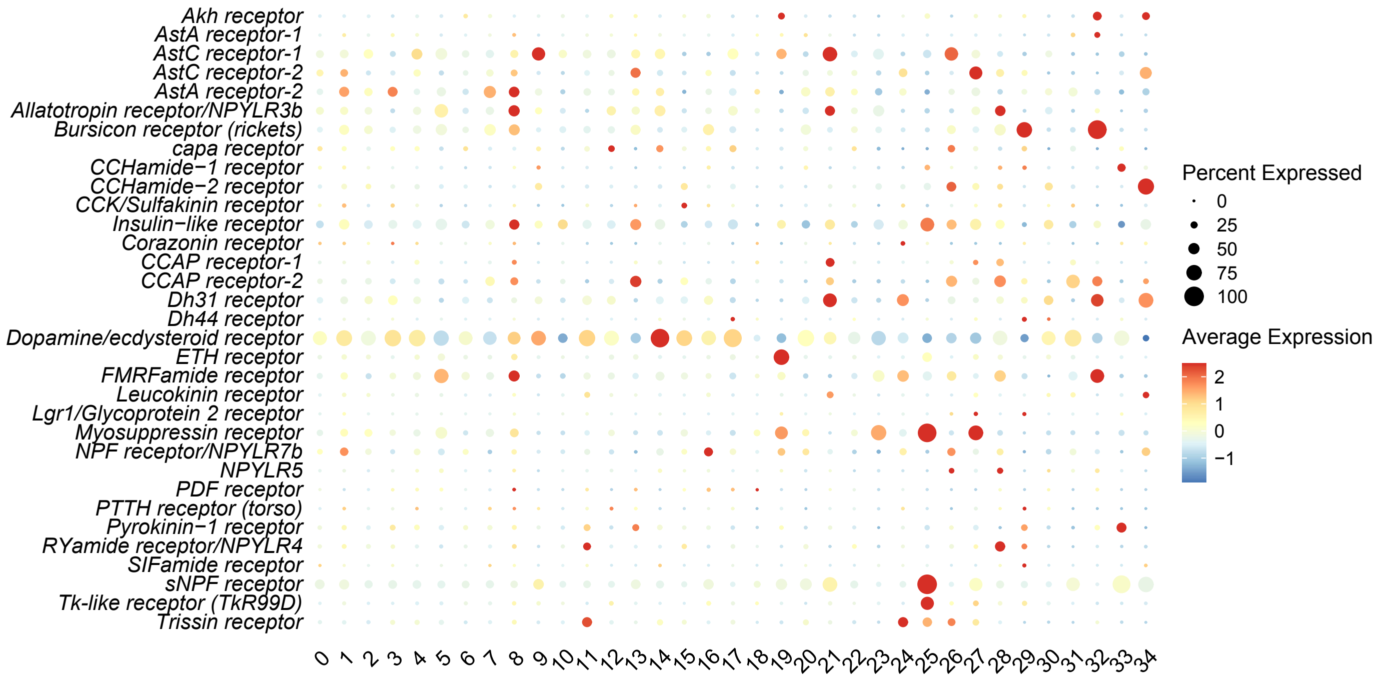

## Figure S13

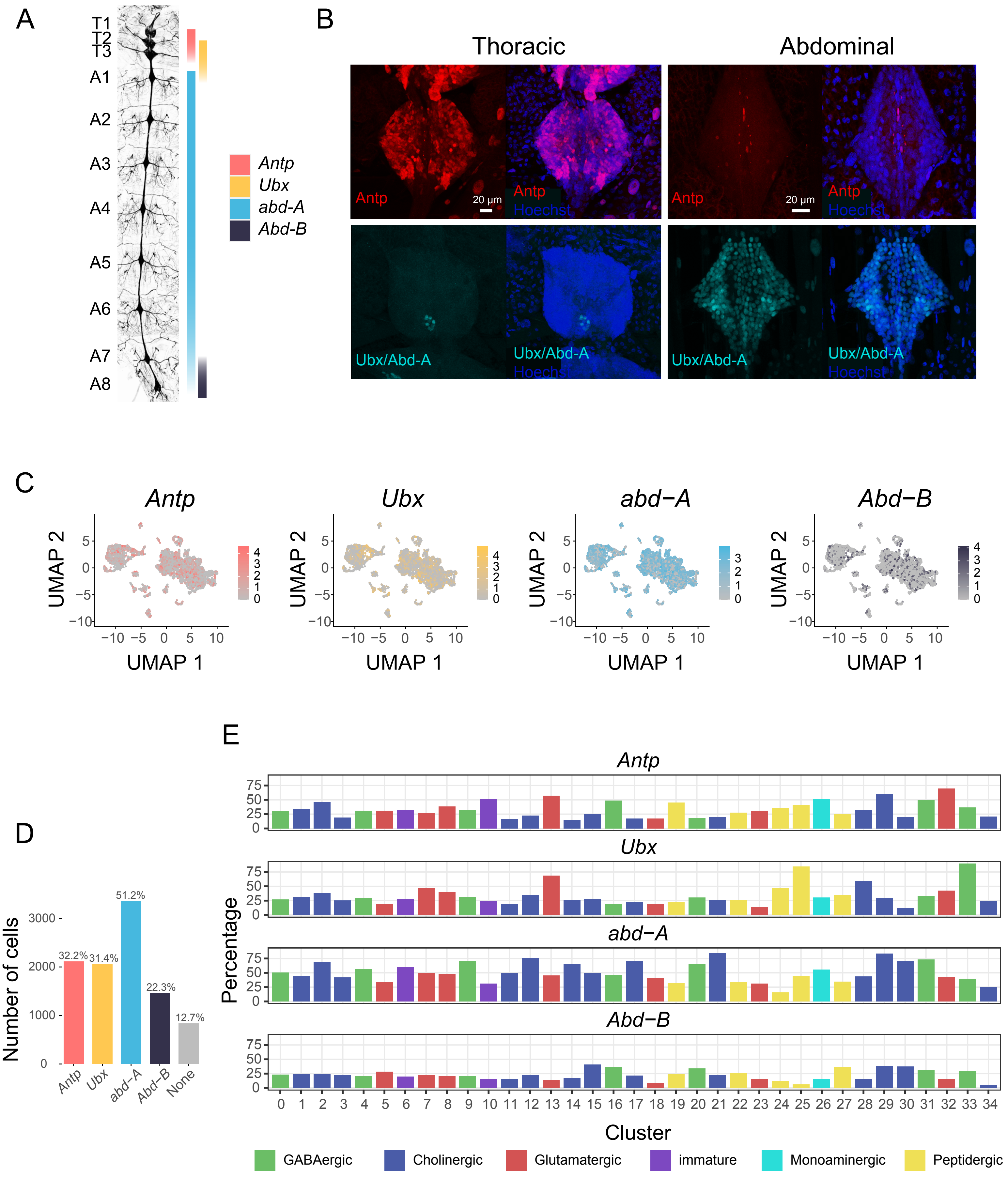

A
T1
T2
T3
A1
A2
A3
A4
A5
A6
A7
A8
Antp
Ubx
abd-A
Abd-B
B
Thoracic
Abdominal
Antp
20 µm
Antp
Hoechst
Ubx/Abd-A
C
Antp
Ubx
abd−A
Abd−B
UMAP 2
UMAP 1
D
Number of cells
32.2%
31.4%
51.2%
22.3%
12.7%
None
E
Percentage
Cluster
GABAergic
Cholinergic
Glutamatergic
immature
Monoaminergic
Peptidergic

## Figure S14

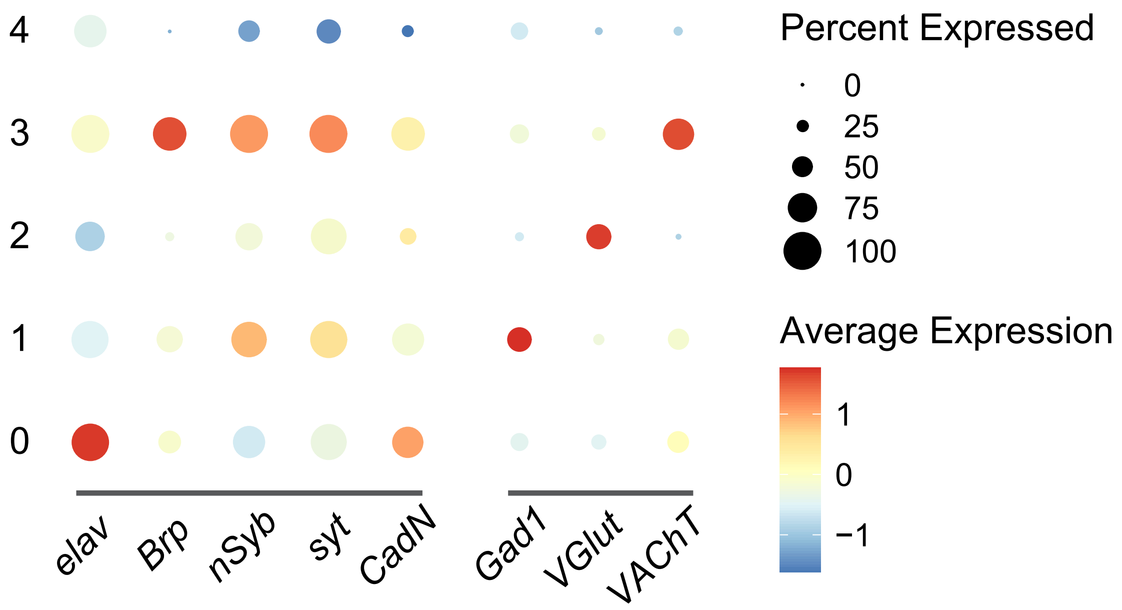

4
3
2
1
0
elav
Brp
nSyb
syt
CadN
Gad1
VGlut
VAChT
Percent Expressed
0
25
50
75
100
Average Expression
1
0
−1

# Figure S15

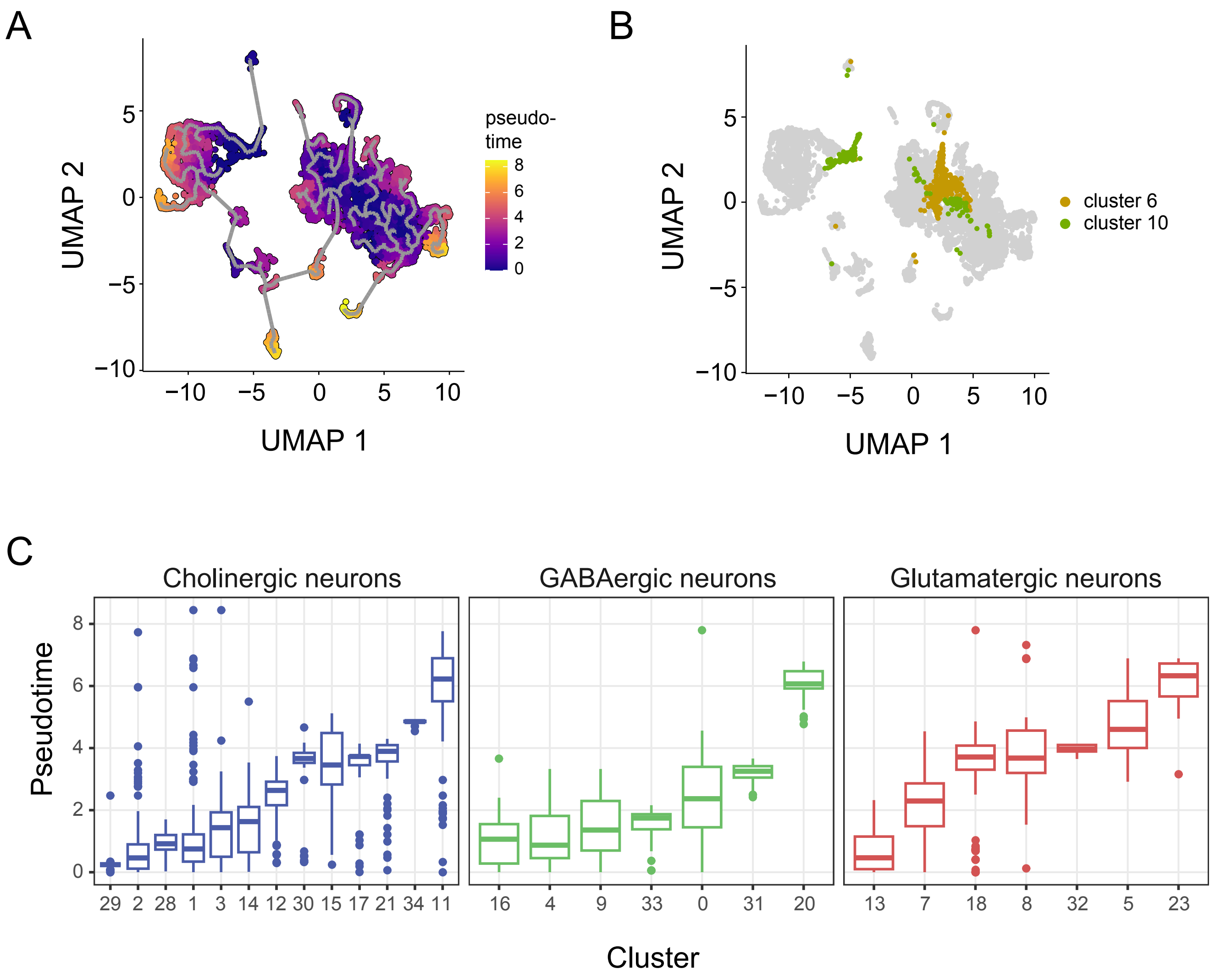
A
B
C
UMAP 2
UMAP 1
pseudo-
time
cluster 6
cluster 10
Cholinergic neurons
GABAergic neurons
Glutamatergic neurons
Pseudotime
Cluster
29 2 28 1 3 14 12 30 15 17 21 34 11
16 4 9 33 0 31 20
13 7 18 8 32 5 23

## Figure S16

A

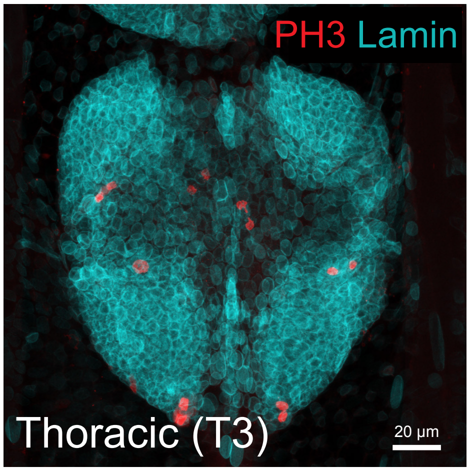


B

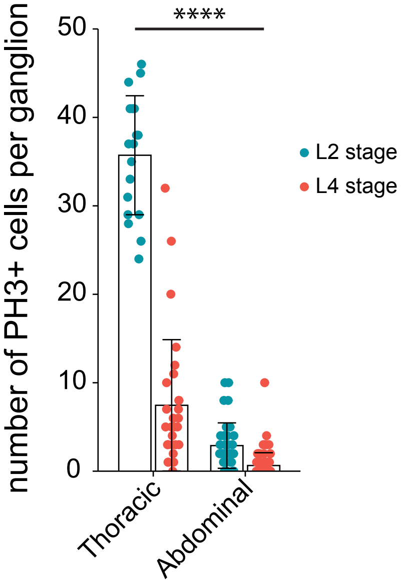

**Figure S17**

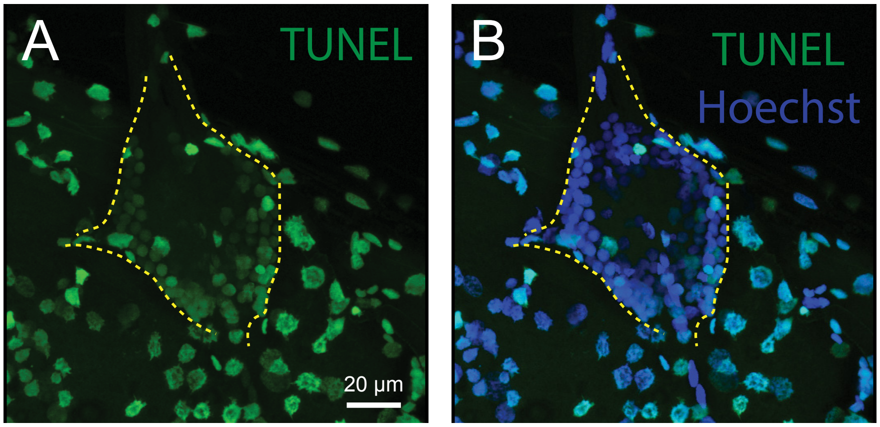

Supplement: Supplementary file 1 — Additional file 1: Fig. S1. Quality control measurements for larval Aedes VNC dataset. (A) Histogram showing the log10 distribution of cells expressing the indicated number of UMIs. Red line represents the nUMI cutoff, with cells below 1000 UMI excluded from further analysis. (B) Histogram showing the log10 distribution of cells with the indicated number of expressed genes (nGene). Red line represents the nGene cutoff; cells with fewer than 500 expressed genes were excluded from further analysis. (C) Histogram showing the distribution of cells with indicated proportion of mitochondrial transcripts. Red line represents the mitochondrial proportion cutoff (18%), above which cells were eliminated from further analysis. (D) Histogram showing the distribution of cells with the indicated proportion of transcripts from ribosomal genes. Cells with ribosomal gene proportions less than 5% or greater than 40% were eliminated from further analysis. Genotype: brp-T2A-QF2w / +; QUAS-mcd8GFP / +. Fig. S2. Identification of putative glial cells in the larval Aedes VNC. (A) Cell atlas from initial clustering, which contains 25 distinct cell clusters. Putative glial cells, identified based on marker gene expression, are indicated. (B) Cells from this putative glial cluster were isolated and reclustered to reveal putative glial subtypes. Feature plots depict expression of glial marker genes including the pan-glial marker repo, cortex glia marker wrapper, surface glia marker gemini and four astrocyte glial markers wunen-2 (wun2), Excitatory amino acid transporter 1 (Eaat1), Gat and glutamine sythetase [57]. Genotype: brp-T2A-QF2w / +; QUAS-mcd8GFP / +. Fig. S3. Distribution of neuronal marker gene expression in the Aedes larval VNC cell atlas. Feature plots depict expression of nSyb, Syt, and CadN which are present in all clusters. Genotype: brp-T2A-QF2w / +; QUAS-mcd8GFP / +. Fig. S4. Neurotransmitter marker gene expression in the larval Aedes VNC. (A-D) Feature plots showing expressi [file 13064_2023_178_MOESM1_ESM.zip › Yin_AedesVNC_suppFigs-revised-v2.pdf]
