# Supplementary material for: A cell atlas of the larval Aedes aegypti ventral nerve cord
Source: Neural Dev. 2024 Jan 31;19:2. doi: 10.1186/s13064-023-00178-8 (PMC10829479; doi:10.1186/s13064-023-00178-8)
Supplement: Supplementary file 1 — Additional file 1: Fig. S1. Quality control measurements for larval Aedes VNC dataset. (A) Histogram showing the log10 distribution of cells expressing the indicated number of UMIs. Red line represents the nUMI cutoff, with cells below 1000 UMI excluded from further analysis. (B) Histogram showing the log10 distribution of cells with the indicated number of expressed genes (nGene). Red line represents the nGene cutoff; cells with fewer than 500 expressed genes were excluded from further analysis. (C) Histogram showing the distribution of cells with indicated proportion of mitochondrial transcripts. Red line represents the mitochondrial proportion cutoff (18%), above which cells were eliminated from further analysis. (D) Histogram showing the distribution of cells with the indicated proportion of transcripts from ribosomal genes. Cells with ribosomal gene proportions less than 5% or greater than 40% were eliminated from further analysis. Genotype: brp-T2A-QF2w / +; QUAS-mcd8GFP / +. Fig. S2. Identification of putative glial cells in the larval Aedes VNC. (A) Cell atlas from initial clustering, which contains 25 distinct cell clusters. Putative glial cells, identified based on marker gene expression, are indicated. (B) Cells from this putative glial cluster were isolated and reclustered to reveal putative glial subtypes. Feature plots depict expression of glial marker genes including the pan-glial marker repo, cortex glia marker wrapper, surface glia marker gemini and four astrocyte glial markers wunen-2 (wun2), Excitatory amino acid transporter 1 (Eaat1), Gat and glutamine sythetase [57]. Genotype: brp-T2A-QF2w / +; QUAS-mcd8GFP / +. Fig. S3. Distribution of neuronal marker gene expression in the Aedes larval VNC cell atlas. Feature plots depict expression of nSyb, Syt, and CadN which are present in all clusters. Genotype: brp-T2A-QF2w / +; QUAS-mcd8GFP / +. Fig. S4. Neurotransmitter marker gene expression in the larval Aedes VNC. (A-D) Feature plots showing expressi [file 13064_2023_178_MOESM1_ESM.zip › Yin_AedesVNC_suppTables-revised.pdf]

Table S1. Antibodies used in this study.

Primary antibodies that yielded specific signal

antibody	antigen	host	source	dilution	distribution
ADL84.12	Lamin	mouse	DSHB	1:500	ubiquitous; nuclear envelope
anti-Elav	Elav	rabbit	Valérie Hilgers	1:500	pan-neuronal
anti-GABA	GABA	rabbit	Millipore Sigma	1:500	GABAergic neurons, enriched in the posterior portion of each ganglion
anti-TH	TH	mouse	Immunostar	1:500	sparse labeling: dopaminergic neurons
anti-serotonin	5-HT	rabbit	Millipore Sigma	1:500	sparse labeling: serotonergic neurons
anti-PH3	PH3	rabbit	Cell Signaling Technology	1:500	labels mitotic cells
anti-CCAP	CCAP	rabbit	Benjamin White	1:500	sparse labeling: peptidergic neurons
anti-Pburs	Pburs	rabbit	Benjamin White	1:100	sparse labeling: peptidergic neurons
2B10	Cut	mouse	DSHB	1:100	labels >50% of VNC neurons; different levels in different neurons
anti-Kn	Knot	guinea pig	Adrian Moore	1:250	sparse labeling; <20 neurons in each ganglion
4D9	en	mouse	DSHB	1:10	sparse labeling of ~20 neurons, enriched at the posterior end in each ganglion
8C11	Antp	mouse	DSHB	1:20	broad labeling at the thoracic ganglion
FP6.87	Ubx/Abd-A	mouse	DSHB	1:20	broad labeling in T3 and abdominal ganglion
40.3A4	Tup/islet	mouse	DSHB	1:50	sparse labeling (but high background); 10-20 cells/ganglion
anti-eve	eve	rabbit	DSHB	1:250	sparse labeling: 3-5 cells per ganglion
ChAT4B1	ChAT	mouse	DSHB	1:25	detected signals at the neuropile
anti-VGLut	VGlut	rabbit	Aaron DiAntonio	1:500	ubiquitously label cell membrane
anti-unc4	unc-4	rabbit	James Skeath	1:500	sparse labeling: 3-5 cells per ganglion
acTub	acetylated microtubules	mouse	sigma	1:1000	ubiquitous (acetylated microtubules)
c1.4	connectin	mouse	DSHB	1:25	weak labeling in neuropile
bp102	CNS axons, drosophila	mouse	DSHB	1:25	weak labeling in neuropile
3C11	synorf	mouse	DSHB	1:25	detected signals at the neuropile
Broad-core (25E9.D7)	broad	mouse	DSHB	1:50	very few cells in CNS
mAbdac1-1	dac	mouse	DSHB	1:5	

Primary Antibodies that yielded no specific signal

antibody	antigen	host	source	dilution
eya10H6_4	eya	mouse	DSHB	1:50
HDC-U33	hdc	mouse	DSHB	1:50
anti-Mira	miranda	guinea pig	Clemens Cabernard	1:50
anti-dpn	dpn	guinea pig	James Skeath	1:500
anti-repo	repo	guinea pig	Benjamin Altenheim	1:500
anti-repo	repo	rabbit	Benjamin Altenheim	1:500
anti-repo	repo	rat	Benjamin Altenheim	1:500
anti-burs	burs	rabbit	Ben White	1:100
anti-DCP-1	DCP-1	rabbit	Cell Signaling Technology	1:100
anti-vg	vg	rabbit	Kirsten Guss	1:100
apterous + collier	ap	rat	Claude Desplan	1:100
1D4	FasII	mouse	DSHB	1:25
Abrupt	Abrupt	mouse	DSHB	1:25
DDA2.7	EcR (common)	mouse	DSHB	1:25
CF.2C7	if	mouse	DSHB	1:25
Acj6	Acj6	mouse	DSHB	1:50
Nub 2D4	nubbin/pdm2	mouse	DSHB	1:50
Tango	Tango	mouse	DSHB	1:3
8B12H9	Yan	mouse	DSHB	1:50
10D3 anti-wrapper	wrapper	mouse	DSHB	1:25
CrebA Rbt-PC	CrebA	rabbit	DSHB	1:3000

Conjugated Antibodies

Antibody	source	dilution
goat anti-HRP-CY3	jackson immuno	1:250
goat anti-HRP-CY5	jackson immuno	1:250
goat anti-HRP-AF488	jackson immuno	1:250
goat anti-chicken AF488	molecular probes	1:250
goat anti-mouse RRX	jackson immuno	1:250
donkey anti-guinea pig RRX	jackson immuno	1:250
goat anti-rabbit RRX	jackson immuno	1:250

Table S3. Proportion of cells in each cluster expressing neurotransmitter marker genes.

cluster	Cholinergic	GABAergic	Glutamatergic	Monoaminergic
0	14.74%	98.18%	4.47%	13.41%
1	91.51%	27.17%	9.06%	26.60%
2	83.61%	20.90%	15.91%	17.58%
3	92.34%	21.29%	4.31%	21.05%
4	17.06%	89.76%	12.60%	31.50%
5	6.09%	15.07%	99.42%	11.59%
**6**	41.69%	24.45%	59.87%	15.05%
7	19.29%	15.43%	98.71%	12.86%
8	24.33%	39.16%	98.48%	13.31%
9	10.95%	97.01%	4.48%	12.44%
**10**	33.83%	25.87%	24.88%	14.93%
11	95.92%	24.49%	4.59%	15.31%
12	96.91%	23.20%	5.15%	14.95%
13	13.33%	20.56%	92.78%	11.67%
14	93.60%	19.77%	8.72%	17.44%
15	86.34%	22.36%	4.35%	14.29%
16	15.09%	98.11%	5.66%	14.47%
17	100.00%	21.15%	3.21%	17.95%
18	17.81%	22.60%	76.03%	5.48%
**19**	14.06%	29.69%	10.16%	22.66%
20	4.46%	93.75%	2.68%	40.18%
21	87.96%	29.63%	10.19%	17.59%
**22**	43.62%	38.30%	18.09%	24.47%
23	8.60%	11.83%	95.70%	8.60%
**24**	17.58%	23.08%	9.89%	7.69%
**25**	9.41%	17.65%	12.94%	7.06%
26	13.16%	30.26%	73.68%	97.37%
**27**	12.33%	27.40%	16.44%	24.66%
28	95.89%	20.55%	8.22%	15.07%
29	70.00%	16.67%	5.00%	1.67%
30	93.22%	23.73%	5.08%	28.81%
31	0.00%	98.08%	3.85%	34.62%
32	10.00%	30.00%	97.50%	50.00%
33	7.89%	100.00%	5.26%	15.79%
34	95.83%	29.17%	0.00%	33.33%

Table S4. Proportion of cells in each cluster expressing marker genes for 0, 1, or multiple neurotransmitters.

cluster	0	1	2	3	4
0	0.33%	71.52%	25.33%	2.65%	0.17%
1	3.21%	49.43%	37.55%	9.43%	0.38%
2	2.38%	62.71%	29.93%	4.51%	0.48%
3	3.59%	59.81%	30.86%	5.50%	0.24%
4	NA	55.64%	38.32%	5.51%	0.52%
5	0.58%	70.72%	24.64%	4.06%	NA
6	0.94%	62.70%	30.72%	5.64%	NA
7	0.64%	61.09%	29.58%	8.68%	NA
8	0.76%	42.21%	39.16%	16.73%	1.14%
9	2.49%	71.64%	24.38%	1.49%	NA
**10**	**25.37%**	52.24%	20.40%	1.49%	0.50%
11	2.55%	58.67%	34.69%	4.08%	NA
12	2.58%	58.25%	35.57%	3.61%	NA
13	4.44%	58.89%	30.56%	6.11%	NA
14	1.74%	62.79%	29.65%	5.81%	NA
15	6.21%	63.35%	27.33%	3.11%	NA
16	1.89%	67.30%	26.42%	4.40%	NA
17	NA	64.74%	28.85%	5.77%	0.64%
18	13.01%	55.48%	28.08%	3.42%	NA
**19**	**41.41%**	44.53%	10.16%	3.91%	NA
20	5.36%	50.00%	42.86%	1.79%	NA
21	5.56%	51.85%	36.11%	4.63%	1.85%
**22**	**19.15%**	44.68%	28.72%	7.45%	NA
23	2.15%	74.19%	20.43%	3.23%	NA
**24**	**54.95%**	32.97%	10.99%	1.10%	NA
**25**	**60.00%**	32.94%	7.06%	NA	NA
26	1.32%	15.79%	55.26%	22.37%	5.26%
**27**	**43.84%**	32.88%	21.92%	1.37%	NA
28	4.11%	64.38%	20.55%	9.59%	1.37%
**29**	**21.67%**	63.33%	15.00%	NA	NA
30	1.69%	57.63%	30.51%	8.47%	1.69%
31	1.92%	59.62%	38.46%	NA	NA
32	2.50%	27.50%	50.00%	20.00%	NA
33	NA	76.32%	21.05%	NA	2.63%
34	NA	45.83%	50.00%	4.17%	NA

Table S6. Aedes aegypti neuropeptide genes.

Neuropeptides	AAEL_ID	LOC_ID	Dro_CGID
Adipokinetic hormone (AKH)	AAEL011996	LOC5575676	CG1171
Adipokinetic/corazonin peptide (ACP)	AAEL010950	LOC5574131	ND
Agatoxin-like neuropeptide	PA	NA	ND
Allatostatin A (ASTA)	AAEL021147	LOC5579498	CG13633
Allatostatin C (ASTC)	AAEL005747	LOC5566967	CG14919
Allatotropin	AAEL009541	LOC5572123	ND
Apis-ITG-like	AAEL006369	NA	CG8216
Bursicon	AAEL026321	LOC110677973	CG13419
Partner of bursicon	AAEL013722	LOC5578519	CG15284
CCHamide 1	AAGE02019353	NA	CG14358
CCHamide 2	AAEL026488	LOC5565599	CG14375
Corazonin	AAEL005252	LOC110673994	CG3302
Crustacean cardioactive peptide (CCAP)	AAEL000630	LOC5564839	CG4910
Diuretic hormone 31 (DH31)	AAEL008070	LOC5569983	CG13094
Diuretic hormone 44 (DH44)	AAEL008292	LOC5570395	CG8348
Ecdysis triggering hormone (ETH)	AAEL001762	LOC5572299	CG18105
Eclosion hormone	AAEL011229	LOC5574558	CG5400
FMRFamide	AAEL013645	LOC5578330	CG2346
Glycoprotein A2	AAEL022126	LOC110676562	CG17878
Glycoprotein B5	AAEL001474	LOC5570903	CG40041
Insulin-like peptide (ILP) 1	AAEL000937	LOC5567485	CG14173
ILP2	AAEL000960	NA	CG8167
ILP3	DQ845751	NA	CG14167
ILP4	AAEL000932	LOC5567486	CG6736
ILP5	AAEL003000	LOC5580310	CG33273
ILP6	DQ845755	NA	CG14049
ILP7	DQ845757	NA	CG13317
ILP8	DQ845754	NA	CG14059
Ion transport peptide	AAEL015332	NA	CG13586
Limostatin 1	AAEL008355	LOC5570460	CG8317
Limostatin 2	AAEL008359	LOC5570459	ND
Kinin (leucokinin)	AAEL010172	LOC5572957	CG13480
Myoinhibitory peptide	AAEL012139	LOC5575871	CG6456
Myosuppressin	AAEL007294	NA	CG6440
Natalisin	AAEL003260	LOC5577647	CG34388
Neuropeptide F	AAEL002733	LOC5575886	CG10342
Neuropeptide-like peptides (NPLPs)	AAEL014708	NA	CG3441
Orcokinin	AAEL010172	LOC5572957	CG13565
Ovary ecdysteroidogenic hormone (OEH)	AAEL004155	LOC5564261	ND
Pigment dispersing hormone	AAEL001754	LOC5572286	CG6496
Proctolin	ND	NA	CG7105
Prothoracicotropic hormone (PTTH)	AAEL026383	LOC110677702	CG13687
Pyrokinin 1 (PK1)	AAEL012060	LOC5575794	CG6371
Pyrokinin 2 (PK2)	AAEL005444	LOC5566490	CG15520
RYamide	AAEL011702	LOC5575249	CG40733
Short neuropeptide F 1 (sNPF1)	AAEL012542	NA	CG13968
Short neuropeptide F 2 (sNPF2)	AF155738.1	NA	ND
SIFamide	AAEL009858	LOC5572527	CG33527
Sulfakinin	PA	NA	CG18090
Tachykinin	AAEL006644	LOC5568206	CG14734

Trissin	AAEL008756	LOC5571024	CG14871
sNPF	AAEL019691	LOC5580248	NA
NPLP	AAEL026044	LOC110681488	NA
allatostatin B	AAEL012139	LOC5575871	NA
partner of bursicon	AAEL013722	LOC5578519	NA
ion transport peptide-like	AAEL019725	LOC5567317	NA
leucokinins	AAEL010172	LOC5572957	NA
cardioactive peptide	AAEL000630	LOC5564839	NA
ASTCC	AAEL024788	LOC110676661	NA
